# The British Association

**Published:** 1905-08-26

**Authors:** 


### The Hospital.

A Journal of

The Medical Sciences and Hospital Administration.

Vol. XXXVIII.—No. 987. SATURDAY, AUGUST 26, 1905.

### The British Association.

The meeting in South Africa this year of the British Association for the Advancement of Science, although it will unquestionably have a stimulating effect upon the intellectual life of the colony itself, and although it must no doubt be regarded as an endeavour to forge a new link in the chain of Imperialism, is yet somewhat to be regretted from a purely domestic point of view. It not only deprives the inhabitants of these islands of a quantity of valuable mental pabulum which they have been accustomed, at this season, to receive; but it also tends to substitute for that pabulum reports of so condensed a character as to render it difficult, in some cases, to do full justice to the authors of papers which are presented in so curtailed a guise, or even to be quite sure that their views have been correctly represented. For example, in the *Times* of last Saturday, we read that, during the sittings in Cape Town, Dr. Mitchell gave an exhaustive paper on "Plague," showing the intimate connection between plague in man and in rodents, especially rats; and that Dr. Black read a paper on "Leprosy," in which he strongly expressed the opinion that the disease was "in no way due to the eating of fish." Dr. Black, we may presume, desired to range himself as an opponent of the views advocated by Mr. Jonathan Hutchinson, who, it must be remembered, has never attributed or attempted to attribute leprosy to the eating of "fish," but solely to the eating of decayed fish, a difference which at once rules out of court an enormous amount of so-called evidence resting upon assertions that certain fish-eating populations are exempt from leprosy. The brief summary which we have quoted throws no light upon Dr. Black's real position; but the question at issue is so serious a one, and affects so large a portion of the earth's surface, and so many of its inhabitants, that it can hardly be passed over without some notice. Mr. Hutchinson, if we rightly understand his views, maintains that the virus of leprosy is only known to us as an associate of piscine decomposition, and that it is only introduced into the human system through the digestive organs; that is to say, either by eating the decomposed fish, or by eating other forms of food which have been contaminated by passing through the hands of lepers, and have thus received their infective properties from direct contact with leprous sores. Mr. Hutchinson has travelled over a large part of the globe for the express purpose of investigating the question, and has visited every population among which leprosy extensively prevails. The missing link in his chain of facts and arguments is that, so far, no one has found the bacillus of leprosy in putrid fish; and the gap thus indicated is, of course, a serious one. It may at any moment be bridged over by some new discovery, and possibly by some new discovery which will materially enlarge the scope of our present knowledge with regard to other phenomena of disease communication. Not having the text of Dr. Black's paper before us, we cannot say, of course, what reservations of expression it may have contained; but, speaking generally, we should not be disposed to concede to any one the right to meet the views of Mr. Hutchinson by the mere expression of an adverse "opinion," however strongly it might be entertained. Those views can only be met by experience as extensive, and by inductions as large, as those by which they are sustained; and it will be a matter of legitimate regret if any member of the medical profession has so far departed from the paths of true science as to be betrayed into dogmatism by the limitations of local or personal experience.

The title and brief account of the paper on rats in relation to plague are calculated to call forth very similar reflections. At the commencement of the existing plague outbreak there was, as we all remember, what might almost be described as a rat scare. We were told not only that the rats of any freshly-infected locality suffered earlier than the human inhabitants, but also that these rats, and the fleas dependent upon them, were in all probability the first carriers of infection to mankind. A note of alarm was sounded with regard to rats on shipboard, and it was declared that their escape to land, at ports of entry, would in all probability be the source of epidemics among the native land rats in the first instance, and among men and women in the fulness of time. The whole of the questions at issue have been made the subjects of careful inquiry on the part of the Local Government Board, and the general result of this inquiry has been to show that the danger arising from rats is very much less serious than at one time was supposed. Professor Klein has confirmed the belief that rats are, indeed, subject to plague, and that they mostly perish quickly when inoculated from the human subject. He has ascertained, however, that some of them survive for a considerable period, or even recover; and the form of plague which is propagated from rat to rat speedily undergoes a remarkable diminution of virulence, or even ceases altogether to be infective. Dr. Theodore Thomson has shown that the statistical facts of plague prevalence on shipboard, as among rats and men, are such as to afford complete support to Professor Klein's conclusions, and to justify the belief that the part taken by the rat in communicating plague to man is much less serious than has been suggested, and that the stringent measures of rat destruction on shipboard, which were at one time recommended, may, as a rule, be safely mitigated in severity. These measures implied considerable detention of the vessels concerned, they were all costly in application, and they could not be applied without the infliction of considerable damage upon many kinds of cargo. It will be a matter for much regret if Dr. Mitchell's paper should give fresh vitality to what was beginning to be regarded rather as a played-out scare.

